# Comparison of oral glucose tolerance test and ambulatory glycaemic profiles in pregnant women in Uganda with gestational diabetes using the FreeStyle Libre flash glucose monitoring system

**DOI:** 10.1186/s12884-020-03325-9

**Published:** 2020-10-19

**Authors:** J. M. Milln, E. Walugembe, S. Ssentayi, H. Nkabura, A. G. Jones, M. J. Nyirenda

**Affiliations:** 1 Non-Communicable Diseases Theme, Medical Research Council/Uganda Virus Research Institute and London School of Hygiene and Tropical Medicine (MRC/UVRI & LSHTM) Uganda Research Unit, Plot 51-59, Nakiwogo Road, P. O. BOX 49, Entebbe, Uganda; 2 Department of Endocrinology and Diabetes, Queen Mary University of London, Mile End Road, London, UK; 3 National Institute for Health and Research (NIHR), Exeter Clinical Research Facility, University of Exeter Medical School, Exeter, UK; 4 Department of Non-Communicable Diseases Epidemiology, London School of Hygiene and Tropical Medicine (LSHTM), London, UK

**Keywords:** Gestational diabetes, Non-communicable disease, Africa, Sub-Saharan Africa, Continuous glucose monitoring, Oral glucose tolerance test, Obstetric medicine

## Abstract

**Background:**

The diagnosis of hyperglycaemia in sub-Saharan Africa (SSA) is challenging. Blood glucose levels obtained during oral glucose tolerance test (OGTT) may not reflect home glycaemic profiles. We compare OGTT results with home glycaemic profiles obtained using the FreeStyle Libre continuous glucose monitoring device (FSL-CGM).

**Methods:**

Twenty-eight women (20 with gestational diabetes [GDM], 8 controls) were recruited following OGTT between 24 and 28 weeks of gestation. All women wore the FSL-CGM device for 48–96 h at home in early third trimester, and recorded a meal diary. OGTT was repeated on the final day of FSL-CGM recording. OGTT results were compared with ambulatory glycaemic variables, and repeat OGTT was undertaken whilst wearing FSL-CGM to determine accuracy of the device.

**Results:**

FSL-CGM results were available for 27/28 women with mean data capture 92.8%. There were significant differences in the ambulatory fasting, post-prandial peaks, and mean glucose between controls in whom both primary and secondary OGTT was normal (*n* = 6) and those with two abnormal OGTTs or “true” GDM (*n* = 7). There was no difference in ambulatory mean glucose between these controls and the 13 women who had an abnormal primary OGTT and normal repeat OGTT. These participants had significantly lower body mass index (BMI) than the true GDM group (29.0 Vs 36.3 kg/m^2^, *p*-value 0.014).

Paired OGTT/FSL-CGM readings revealed a Mean Absolute difference (MAD) -0.58 mmol/L and Mean Absolute Relative Difference (MARD) -11.9%. Bland-Altman plot suggests FSL-CGM underestimated blood glucose by approximately 0.78 mmol/L.

**Conclusion:**

Diagnosis of GDM on a single OGTT identifies a proportion of women who do not have a significantly higher home glucose levels than controls. This raises questions about factors which may affect the reproducibility of OGTT in this population, including food insecurity and atypical phenotypes of diabetes. More investigation is needed to understand the suitability of the OGTT as a diagnostic test in sub-Saharan Africa.

## Background

The burden of hyperglycaemia in pregnancy in sub-Saharan Africa (SSA) is not clear, however several studies report a prevalence comparable to the well-studied high-income setting [1]. Indeed, our recent study in urban and peri-urban central Uganda supports this (Natamba B, unpublished). These prevalence estimations are made using the WHO 2013 guidelines which recommend the following criteria for making a diagnosis of hyperglycaemia in pregnancy using the 2-h oral glucose tolerance test (OGTT); fasting ≥5.1 and 2-h ≥ 8.5 mmol/L for gestational diabetes (GDM), and ≥ 7.0 and 2-h ≥ 11.1 mmol/L for diabetes in pregnancy (DIP) [2]. However, these strict glycaemic cut-offs were extrapolated from the multinational HAPO study which did not include women in Africa and has not been validated in the sub-Saharan African setting [3].

Ideally the OGTT should reflect a woman’s ambulatory blood glucose levels, which in turn should predict the chance of poor maternal and perinatal outcomes. There is concern that a number of factors which are more common in the sub-Saharan African setting may lead to misleading OGTT results. For example, food insecurity resulting in low dietary carbohydrate content [4, 5], or a missed or reduced meal size the night preceding the test [6] have all been shown to induce spurious post-OGTT hyperglycaemia. Additionally, physical exertion to reach antenatal clinic, which may reduce fasting glucose results, is common.

Ambulatory continuous glucose monitoring provides an opportunity to measure a woman’s glycaemic profile in her home environment [7]. Abbott’s FreeStyle Libre flash continuous glucose monitoring (FSL-CGM) system provides accuracy similar to currently available blood glucose meters. However, these validation studies have generally been conducted in populations of European descent in the high-income setting with diabetes outside of pregnancy [8–11]; FSL-CGM use in pregnant women in sub-Saharan Africa has not been reported.

The aim of this investigation was to assess how a diagnosis of GDM, based on OGTT results, predicts glycaemic variables in the home environment. We compare OGTT results to mean ambulatory glucose, and discrete fasting and post-prandial periods at home. We also comment on the usability and accuracy of the device using paired OGTT and FSL-CGM measurements.

## Methods

### Study design

This prospective investigation was a nested study within a larger study of gestational diabetes (unpublished) which screened 3852 women with OGTT attending antenatal clinics in Central Uganda. Women for this nested study were recruited from four sites (Two public; Kawempe National Referral Hospital, Entebbe Regional Referral Hospital. Two private; St Francis Hospital Nsambya, Uganda Martyrs Hospital Lubaga) between 16th August 2018 and 24th May 2019.

The study was performed according to Good Clinical Practice and the Principles of the Declaration of Helsinki. The study protocol was approved by Uganda Virus Research Institute Research and Ethics Committee (approval GC/127/19/04/625), the Uganda National Council of Science and Technology (approval HS2340), and local hospital Institutional Review Boards.

### Participants

A total of 28 women who attended antenatal clinic between 24 and 28 weeks were invited to take part, based on the results of their initial OGTT in the study. Twenty cases of ‘GDM’ were included; eleven had high fasting glucose levels (> 5.1 mmol/L) and nine had high 2-h OGTT levels (> 8.5 mmol/L) as per the WHO 2013 criteria for a diagnosis of GDM. Eight ‘Controls’ with normal glucose profiles on initial OGTT were selected for comparison as controls. Baseline demographic and anthropometric characteristics, as detailed below, were collected as part of the main study using standard equipment and procedures.

### Study procedures

Women were fitted with the FSL-CGM device (Freestyle Libre Flash glucose monitoring system, Abbot Diabetes Care, Alameda, California, USA) on the back of the upper arm by study site workers, and given instructions to scan the device at least every 8 h. Women keep a diary of their mealtimes at home indicating the time they consumed either small snacks or large meals. We aimed to collect 48–96 h of ambulatory data starting at least 12 h after the sensor instalment. Women were then invited to return for a second OGTT whilst the device remained in place to obtain paired measurements with reference venous blood glucose.

OGTT was performed as follows: a fasting venous blood glucose level was taken before participants were given 82.5 g glucose monohydrate (equivalent to 75 g anhydrous glucose) dissolved in 250mls of water. Further venous blood samples were taken after 1 and 2 h to measure serum glucose levels. The time was recorded at each blood draw. Samples for glucose measurement were immediately centrifuged, and plasma kept on ice. All samples were analysed centrally at the MRC/UVRI and LSHTM Clinical and Diagnostics Laboratory in Entebbe, within 4 h of collection or stored at − 80 °C for subsequent analysis.

### Statistical procedures

#### Ambulatory glucose profiles of women with GDM versus controls

Women were divided into four groups based on the results of their two OGTTs.

i) Those with two normal OGTTs were labelled as ‘Controls – true’;
ii) those with a normal primary OGTT and abnormal repeat OGTT were labelled as ‘Controls – progressed’;
iii) those with an abnormal primary OGTT and normal repeat OGTT were labelled as ‘GDM – normalised’;
iv) those with two abnormal OGTTs were labelled as ‘GDM – true’.

The ambulatory data from the FreeStyle Libre was downloaded and analysed using the EasyGV© Software Version 9.0.R2 [12] to provide mean and standard deviation of home glucose levels. Participants were included in the ambulatory CGM analysis if the device recorded > 48 h of ambulatory data and with > 80% of total data captured. Missing data were interpolated using the straight-line estimation function of the EasyGV© software. Differences in mean glucose and variability (by standard deviation) between the groups were calculated with a two-sided t-test using STATA® statistical software version 15.1 (College Station, Texas).

Fasting and 2-h glucose levels from the second OGTT were compared to 1) overall mean ambulatory glucose captured with FSL-CGM and 2) fasting and post-prandial periods at home. Fasting periods were identified as > 8 h from the previous meal time, and a maximum of 8 data points (equating to 2 h) were accepted before the first meal taken. Post-prandial glucose excursions were identified 1–3 h after a large meal was taken. The mean of three glucose measurements was taken; the peak level, and the two levels 15 min either side.

#### Performance of the Freestyle libre device

The accuracy of the device was determined by comparing the venous blood glucose result from the repeat OGTT and the most immediate FSL-CGM result that followed, to account for the reported time lag between changes in blood and interstitial blood glucose [13]. Correlation is described using Pearson’s correlation coefficient, Mean absolute relative difference (MARD) and Mean absolute difference (MAD). We provide percentage of paired results satisfying the ISO 15197:2013 criteria [14] which stipulates that 95% of results from the technology under investigation display a MAD of ±0.83 mmol/L (< 15 mg/dL) when reference glucose < 5.5 mmol/L (< 100 mg/dL) or MARD ±15% when reference glucose values > 5.5 mmol/L. Results are plotted on the standard Bland-Altman plot [15].

## Results

### Participants characteristics

The characteristics of the 28 women recruited into the study are displayed in Table 1, divided into the ‘GDM’ and ‘Controls’ groups identified by the initial OGTT.

**Table 1:** Baseline characteristics of study participants

	Mean ± SD	
*n = 28*	***GDM*** *(n = 20)*	***Controls*** *(n = 8)*
Age (years)	29.8 ± 6.7	26.5 ± 4.7
Parity	2.0 ± 1.1	1.3 ± 1.0
Gestation (weeks^+days^)	26^+ 2^ ± 1^+ 3^	26^+ 3^ ± 1^+ 1^
Mid-gestational BMI (kg/m^2)a^	31.5 ± 6.7	31.1 ± 5.2
Haemoglobin (g/dL)	12.4 ± 1.1	12.3 ± 0.9
HbA1c (%)	5.1 ± 0.5	4.9 ± 0.6
HbA1c (mmol/mol)	32.2 ± 5.9	30.0 ± 6.1
Public:private hospital	8:12	3:5

^a^*BMI* (Body Mass Index) at recruitment at 24–28 weeks gestation; *HbA1c* glycosylated haemoglobin

#### Ambulatory glucose profiles of women with GDM versus controls

Of the 28 participants, sufficient ambulatory data were collected on 27 women, with median duration 72 h (range 48–96 h) and mean (±SD) data capture 92.8% (±4.6). Missing data clustered around midnight as night-time presented the longest time period between scanning of the sensor which stores a maximum of 8 h of data. One participant’s data were excluded due to poor data capture (< 80%). The mean number of data points to inform the mean (±SD) ambulatory fasting figure was 21 (±6), and mean (±SD) number of post-prandial peaks was 5 (±2).

The ‘Controls’ group and ‘GDM’ group were divided based on the results of their repeat OGTT; six women had two normal OGTTs (‘Controls – true’), one women had a normal initial OGTT and abnormal repeat OGTT (‘Controls – progressed’), 13 women had abnormal initial OGTTs and normal repeat OGTTs (‘GDM – normalised’), and seven women had two abnormal OGTTs (‘GDM – true’).

**Fig. 1:**
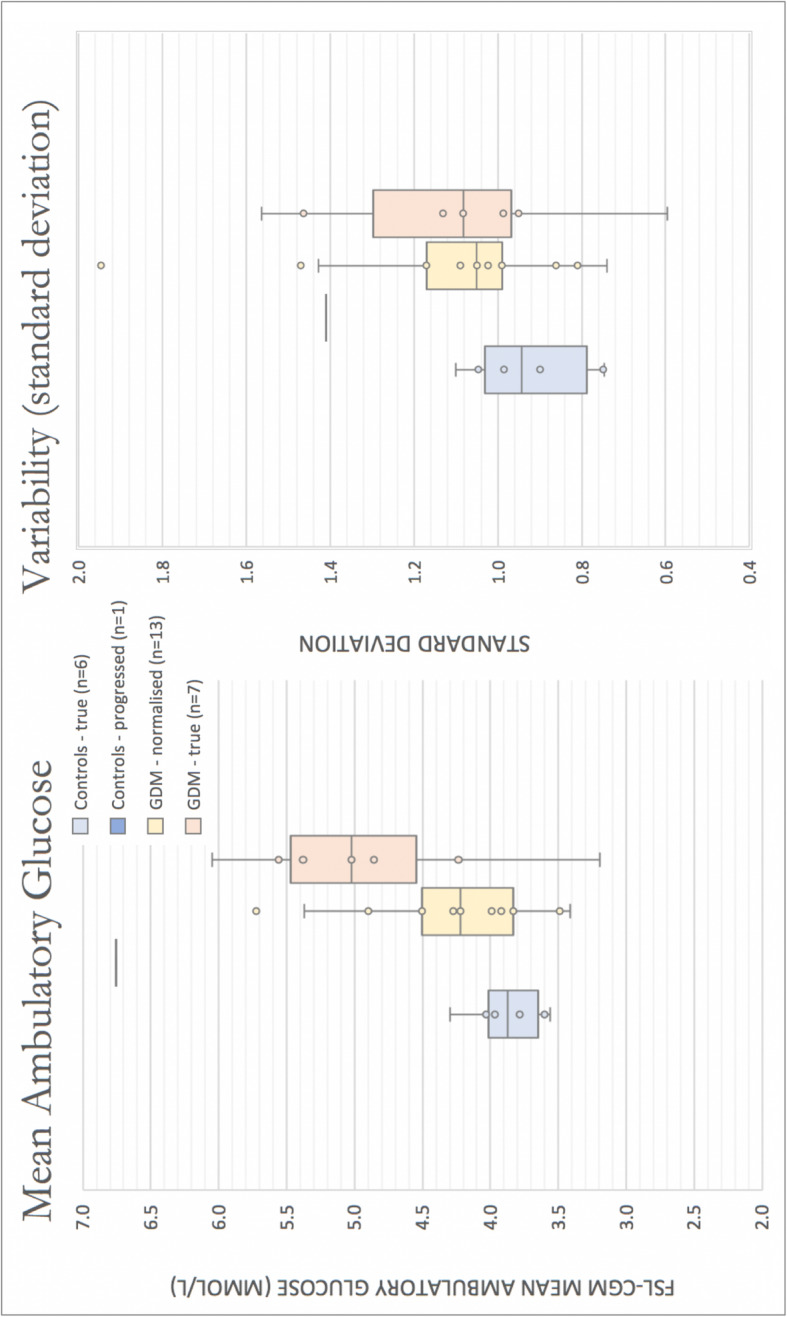
Boxplot showing distribution of mean ambulatory glucose and variability (standard deviation) derived from FSL-CGM results for the four groups. Boxes represent interquartile range, whiskers represent variability, and horizontal bars represent the median value. Individuals are presented as dots. Light blue = ‘Controls – true’; Dark blue = ‘Controls – progressed’; Yellow = ‘GDM – normalised’; Red = ‘GDM – true’

The mean ambulatory glucose levels, variability (standard deviation), and fasting/post-prandial periods captured by FSL-CGM for the four groups are displayed in Table 2 and Fig. 1. Compared with ‘Controls – true’, there was a significant difference (*p* = < 0.05) in the mean ambulatory glucose (*p* = 0.027), fasting periods (*p* = 0.024), and post-prandial peaks (*p* = 0.041) of women identified as ‘GDM – true’. There was no difference in the mean glucose, variability, and fasting periods of the women identified as ‘GDM – normalised’, though there was a significant difference in the post-prandial peaks (*p* = 0.003). These participants whose OGTT normalised had significantly lower mid-gestational BMI than those who had confirmed GDM on two OGTTs (BMI 29.0 Vs 36.3, *p*-value 0.014).

**Table 2:** Differences in ambulatory glucose measurements of participants in the four groups

	Controls	Controls	GDM	p-value*	GDM	p-value*
	True	Progressed	Normalised		True	
Total *n* = 27	*n* = 6	*n* = 1	*n* = 13		*n* = 7	
**Mean glucose (mmol/L)**	3.87	6.75	4.26	*0.22*	4.90	***0.03***
Standard deviation	0.28	–	0.71		0.95	
**Mean variability (SD)**	0.92	1.42	1.13	*0.42*	1.11	*0.22*
Standard deviation	0.15	–	0.25		0.33	
**Fasting periods (mmol/L)**	3.27	6.05	3.40	*0.63*	4.26	***0.02***
Standard deviation	0.40	–	0.58		0.85	
**Post-prandial peaks (mmol/L)**	5.38	9.27	6.44	***< 0.01***	6.82	***0.04***
Standard deviation	0.46	–	0.69		1.46	

*compared with ‘Controls – true’ group

#### Performance of the Freestyle libre device

22/28 women completed the full time period without problems with the FSL-CGM device. Of the remaining six; three sensors were accidently removed (one trapped in a door, another dislodged whilst washing, another with no trauma), two showed an ‘error’ message, and one was removed on request of her partner. We obtained matched FSL-CGM/OGTT data for 17 women out of the 28 who completed a repeat OGTT whilst wearing the device. Of the remaining eleven; six had technical issues as stated above, two sensors were not scanned post-OGTT due to staff error and so data was lost, and three women had their repeat OGTT immediately after the FSL-CGM device was fitted, before the protocol was revised; these data were excluded due to known sensor inaccuracy at this time [13].

**Fig. 2:**
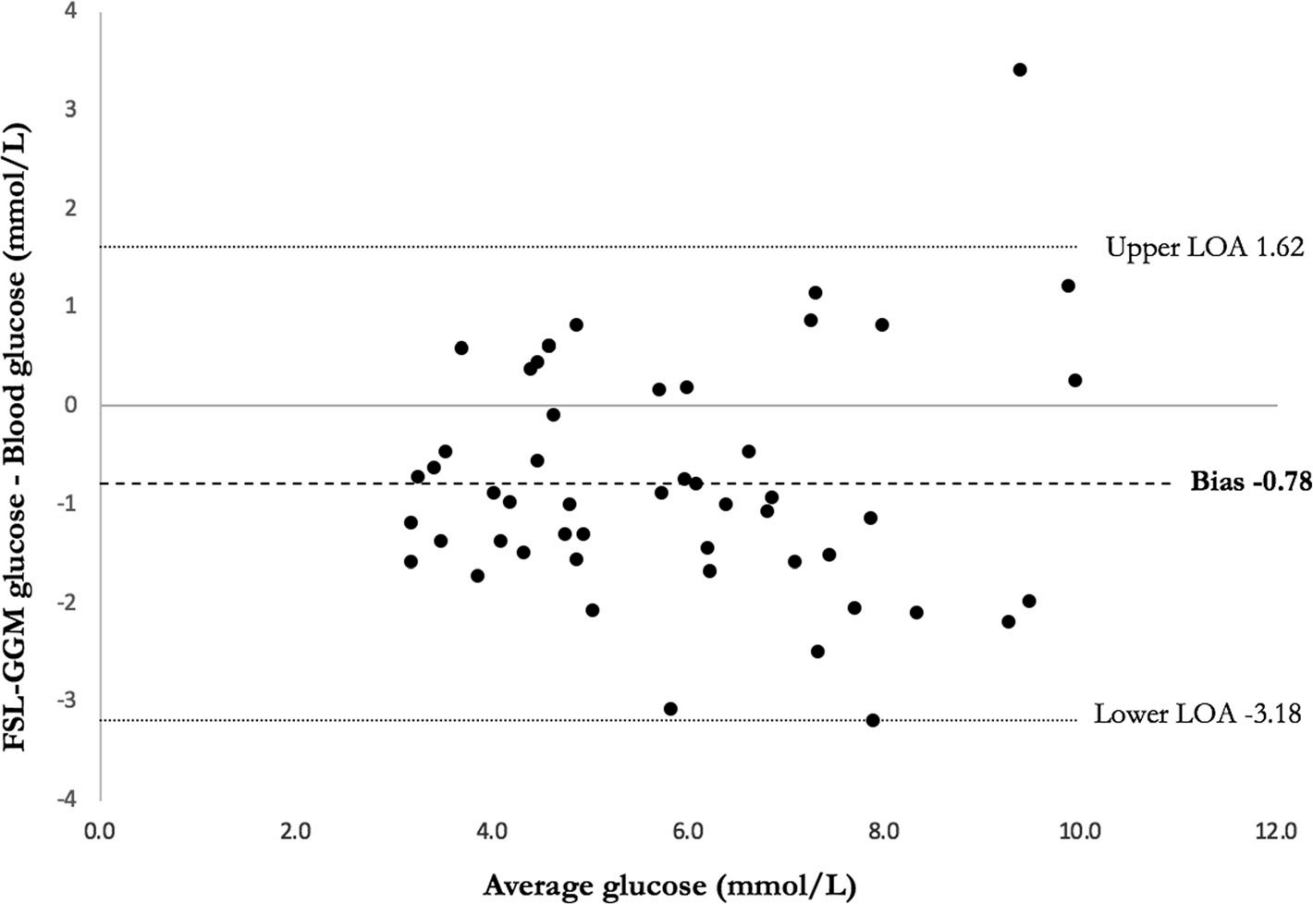
Bland-Altman plot comparing interstitial glucose with FSL-CGM and reference venous blood glucose. Overall FSL-CGM underestimated blood glucose by 0.78 mmol/L, with 95% limits of agreement (LOA) from − 3.18 to 1.62 mmol/L.

The relationship between FSL-CGM interstitial glucose and venous blood glucose during the OGTT is shown in Table 3. The mean (±SD) delay between the venous blood glucose result and the interstitial FSL-CGM result was 7.4 (±5.3) minutes. The overall correlation (95% CI) between CGM glucose and venous glucose for combined fasting, 1-h and 2-h results was 0.81 (0.69–0.89). Overall 49% results met the ISO 2013 criteria, using MAD for the 21/51 results < 5.5 mmol/L and MARD for the 30/51 results > 5.5 mmol/L. Bland-Altman plot is displayed in Fig. 2.

**Table 3:** Comparison of interstitial glucose measured by FSL-CGM and reference venous blood glucose based on standard analytical approaches

	Fasting (*n* = 17)	1 h post-OGTT (*n* = 17)	2 h post-OGTT (*n* = 17)	All (*n* = 51)
Time difference between paired samples ± SD (minutes)	8.8 ± 5.4	5.3 ± 5.0	7.9 ± 5.2	7.4 ± 5.3
Correlation coefficient (95% CI)	0.57 (0.12–0.83)	0.68 (0.30–0.88)	0.66 (0.26–0.87)	0.81 (0.69–0.89)
MAD overall (mmol/L)	−0.54	− 0.92	−0.89	− 0.78
MAD when ref. < 5.5 mmol/L (*n* = 21)				−0.58
MARD overall (%)	−12.2	−11.3	−13.2	−12.3
MARD when ref. > 5.5 mmol/L (*n* = 30)				−11.9
Within ISO 2013 criteria (%)	58.8	41.2	47.1	49.0

*SD* standard deviation; *CI* confidence interval; *MAD* mean absolute difference; *MARD* mean absolute relative difference; *ISO* international organization of standardization

## Discussion

### Summary

Our study describes potential shortfalls of the OGTT when used in the sub-Saharan African setting. Whilst the OGTT successfully predicted higher ambulatory glycaemic values in women consistently diagnosed with GDM on two OGTTs, there was a significant proportion of women with only one abnormal OGTT and who did not display significant elevations in ambulatory mean glucose levels. However, these women still had significantly higher home post-prandial glycaemic excursions than controls, suggesting that they may have subtle abnormalities in glucose control. Moreover, these women were of lower BMI compared to the true GDM group. These observations indicate challenges in reproducibility of OGTT in this population, particularly in individuals with low BMI.

The FreeStyle Libre flash glucose monitoring (FSL-CGM) technology was highly acceptable to pregnant women living in Kampala, Uganda. However, attrition rate due to sensor loss was high. The device modestly underestimates venous blood glucose and displays a higher mean average deviation than initial accuracy studies of the device under laboratory conditions; this will require further investigation in similar populations.

#### Ambulatory glucose profiles of women with GDM versus controls

In our study, the OGTT successfully predicted higher mean ambulatory glucose levels, fasting levels, and post-prandial peaks in women with GDM confirmed on repeat OGTT testing. We are not aware of any other studies that make such a comparison, particularly not in this setting. The majority of studies in pregnancy have been designed to test the utility of CGM devices in women in high-income countries (HICs) already diagnosed with type 1 or type 2 diabetes to reduce glycaemic indices such as HbA1c and poor perinatal outcomes [16–19]. A small study in patients with cystic fibrosis [20] compared OGTT with CGM results, and found some overlap of glucose levels in those with normal OGTT results and those deemed to have impaired glucose tolerance, as we did in our study. A small study in Japan [21] compared CGM results in women diagnosed with GDM on 1 h and 2 h OGTT results and found a higher SD in glycaemic profiles, though they only included two controls. Both these studies used the Medtronic iPro™2 technology, and not the FreeStyle Libre as we did.

A large proportion (13/20) of our participants initially diagnosed with GDM had normal OGTTs despite three further weeks of gestation and only basic dietary advice. They had similar mean glucose levels, variability and fasting levels to the true control group. However, they displayed significantly higher post-prandial peaks at home. This might suggest that the OGTT, using the new stricter WHO 2013 diagnostic criteria, may be over-sensitive in diagnosing GDM in women who do not have significantly elevated mean glucose levels at home. Alternatively, it could be argued that there had been improvement in glycaemic control, perhaps from lifestyle changes after the initial diagnosis; one could also argue this is offset by increasing gestation of an average of 3 weeks. However, this discrepancy may also relate to challenges of OGTT reproducibility in a population where food insecurity remains important. Indeed, there is increasing evidence that the OGTT may be affected by carbohydrate intake during the day(s) preceding the test [4–6]. Secondly this may be a clue that the women falsely identified as GDM may be reflective of an atypical phenotype of diabetes more commonly found in sub-Saharan African populations associated with setting-specific causes of low beta-cell reserve such as in-utero and childhood malnutrition, and inflammation related to chronic infections [22, 23]. Notably, the participants whose OGTTs normalised had significantly lower mid-gestational BMI than those who had confirmed GDM. However, this theory does not explain the normalisation of the OGTT on repeat testing.

#### Performance of the Freestyle libre device

We are aware of one other study reporting real-world usability of the FSL-CGM in sub-Saharan Africa, in Johannesburg, South Africa [24]. They describe similar logistical issues to our own report in that 17/73 (23%) of participants encountered either sensor failure or early removal of the sensor, comparable to our own 6/28 (21%). This compares to 0/73 in a study in pregnant women in England [25] and 49/80 sensor loss in a study in Beijing [26] though this was over a full 2 week period. We postulate this may be secondary to environmental conditions or the physical nature of domestic life in Uganda.

We applied standard metrics for assessment and comparison with other studies [27]. We report similar MAD (0.58 mmol/L) and MARD (11.9%) values to the original performance papers [9, 10, 25, 26, 28] though the last two studies used capillary rather than venous blood glucose as a reference comparator. However, variable accuracy is hidden within these mean values; we report inferior accuracy with lower percentage of values fulfilling ISO criteria (49% Vs 73.2% [10]). A clear difference is that these other studies were investigating people with established diabetes, with higher glucose concentrations. However, the discrepancy in accuracy still remains when looking at the sub-group analysis of accuracy at lower glucose concentrations too. Our Bland-Altman plot describes the tendency of FSL-CGM to underestimate venous blood glucose in our study. This reflects other published data which describes this technology reading ‘low’ during eu- or hypoglycaemia [10, 13], particularly at night.

### Strengths

Our study is the first to compare OGTT results to ambulatory glycaemic profiles in this setting, which is more likely to reflect the situation in other low-income countries where women take part in physical domestic duties and still consume a traditional diet. It is also the first study to assess this technology in the environmental conditions of SSA, with temperatures above the product storage specification (Abbott advise < 25 degrees Celsius [29]) and similar levels of physical domestic life not reflected under previous strict trial conditions.

Secondly, as this was a nested study within a large investigation of hyperglycaemia in pregnancy, we have taken advantage of robust study protocols and laboratory assessment. Samples from different study sites were handled appropriately with immediate local centrifugation and rapid analysis at the MRC/UVRI and LSHTM central laboratory which maintains strict quality control standards. A strong system for analysing the reference sample is imperative to any study, particularly when commenting on performance and accuracy of new technology.

### Weaknesses

The study was limited by the small number of participants involved. A larger study would be needed to make strong conclusions about the ability of the OGTT to predict ambulatory glycaemic load, though we offer important insights. Again, accuracy studies would also benefit from a larger number of participants as was done in previous performance studies of the technology. It should be noted that the study was not specifically designed to test the reproducibility of the OGTT.

Our results may be affected by our assessment during time periods where the technology is reported to exhibit diminished accuracy, namely hypo- (< 4.0 mmol/L) or euglycaemia (4.0-10 mmol/L) [10, 13]. The GDM cases we selected were ‘mild’ and displayed relatively normal glycaemic profiles for much of the day, and so accuracy may be diminished compared to the other studies assessing higher glucose concentrations in overt diabetes. We found many results FSL-CGM displaying ‘2.8 mmol/L’, the lower limit of the sensor, particularly overnight. These are unlikely to be true values, rather the technology displaying an inaccurate underestimation which may be due to compression of the sensor as described elsewhere. Whilst unlikely to be accurate values they would have affected the results of mean glucose burden in some of our participants. Our accuracy assessment may also have been hampered by taking measurements during the OGTT as previous studies suggest that accuracy of the device is also diminished during rapid changes in glucose level [11, 30]. One might argue it is not fair to comment on accuracy solely during an OGTT, and it may be valuable to assess accuracy with paired glucose results throughout the day. Though this may provide a fairer assessment of performance, it would demand extended periods of time for women to attend the study site.

### Future work

The ultimate question remains; what test, or combination of tests, should be deployed to optimise detection of hyperglycaemia in pregnancy in sub-Saharan Africa? The OGTT is an expensive and laborious test to use in the resource limited setting, and its use with the WHO 2013 diagnostic criteria must be adequately studied before being rolled out in this population. More work is needed to understand whether our results represent improvement in glycaemic indices with basic dietary advice, or indeed unreliability of the OGTT in this population. We also do not yet know whether women with normal OGTT but high glucose excursions at home suffer worse pregnancy outcomes.

This is the first reported use of this technology in sub-Saharan Africa, other than South Africa. We await to see if our findings are reproduced in other populations, or with other CGM technology, and methods by which we may reduce sensor attrition rates in this setting.

## Conclusion

New CGM technology offers the opportunity to assess the glycaemic profiles of pregnant women with GDM in everyday life. We describe potential inadequacies of the OGTT to diagnose GDM in the sub-Saharan African setting, and suggest this may be due to physical and dietary conditions around the time of the OGTT, or the existence of atypical phenotypes of diabetes more common in this setting. Our team is in the process of conducting other studies to further test these hypotheses.

## Data Availability

The datasets used and/or analysed during the current study are available from the corresponding author on reasonable request.

## Abbreviations

SSA: Sub-Saharan Africa
OGTT: Oral glucose tolerance test
FSL-CGM: FreeStyle Libre continuous glucose monitoring device
GDM: Gestational diabetes
BMI: Body mass index
MAD: Mean absolute difference
MARD: Mean absolute relative difference
SD: Standard deviation
HbA1c: Glycosylated haemoglobin
ISO: International organization for standardization
LOA: Limits of agreement
HIC: High-income country
DIP: Diabetes in pregnancy
